# Fibrous Hamartoma of Infancy in the Scrotum

**DOI:** 10.1055/s-0039-1697924

**Published:** 2019-11-28

**Authors:** Hrvoje Stepančec, Zoran Kokot, Draženko Keretić, Sandra Radiković, Donat Grgurović

**Affiliations:** 1 Department of Pediatric Surgery, Opca Bolnica Varazdin, Varazdin, Croatia; 2 Department of Pathology, Cithology and Forensic Medicine, Opca Bolnica Varazdin, Varazdin, Croatia; 3 Department of Plastic, Aesthetic and Reconstructive Surgery, Opca Bolnica Varazdin, Varazdin, Croatia

**Keywords:** fibrous, hamartoma, infancy, tumor, scrotum

## Abstract

Fibrous hamartoma of infancy is a solid benign tumor of the subcutis, which usually occurs within the first 2 years of life. It predominantly occurs in males, and is clinically presented as a solid, painless, well-limited subcutaneous formation, tending to grow, and in most cases without any symptoms. It occurs in various locations. The aim of this case report was to present a case of a rare tumor of infancy in the scrotal region, in an 8-month-old male infant, with a nonspecific clinical picture, suggestive of a malignant formation, thus presenting a diagnostic challenge for a doctor. The tumor was completely removed. The diagnosis was confirmed by histopathological analysis. One year after the surgical procedure, a follow-up ultrasonography examination showed no relapse.

## Introduction

**Fig. 1:**
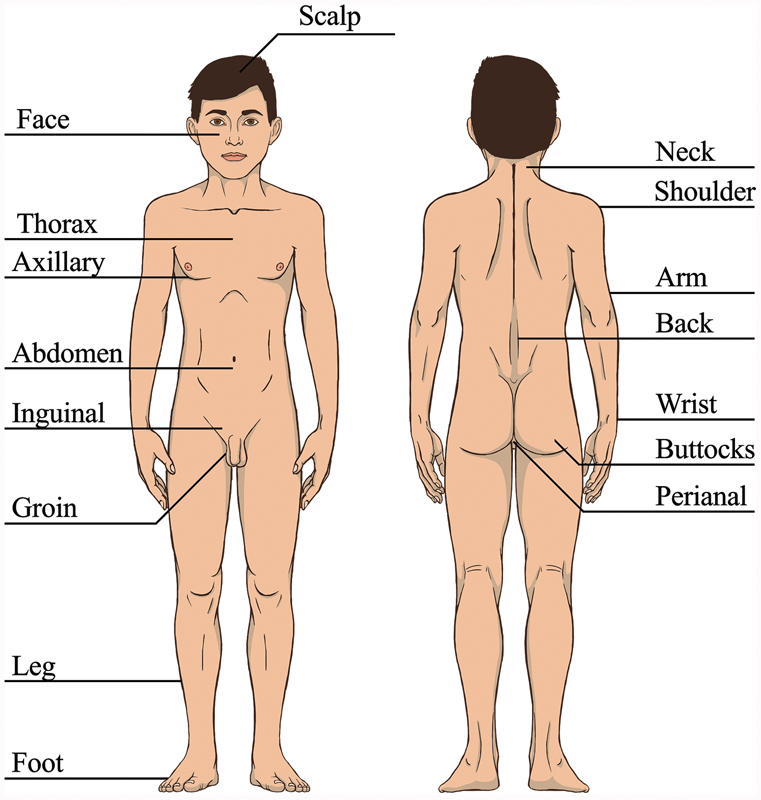
Fibrous hamartoma appears in various places. The scheme presents the most frequent locations.

Fibrous hamartoma of infancy (FHI) is a solid benign tumor of the subcutis, which usually occurs within the first 2 years of life. It was first described by Reye in 1956, as the dermal fibromatous tumor of infancy, and later, within a larger study, Enzinger in 1965 renamed it into fibrous hamartoma of infancy. It accounts for 0.02% of all benign soft tissue tumors. 1 As a congenital entity, it occurs in 15 to 20% of all cases. 2 3 It predominantly occurs in males in the ratio of 2:1. 1 3 4 Clinically, it is presented as a solid, painless, well-limited subcutaneous formation, tending to grow. 5 In most cases, it has no symptoms, so patients report to a doctor only when parents notice a palpable formation. It occurs in various locations in the body, most frequently in the upper arm and the axillary region 2 5 6 ( Fig. 1 ). We report on a child with an FHI of the scrotum.

## Case Report

**Fig. 2:**
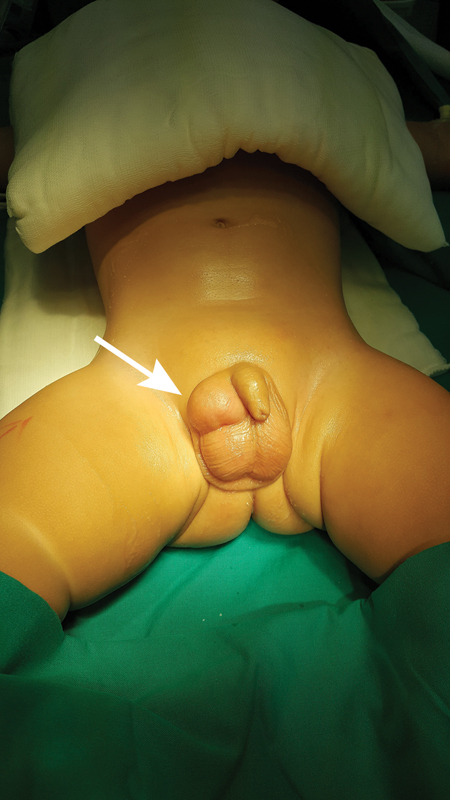
Tumor in the upper half of the right hemiscrotum marked by an arrow.

**Fig. 3:**
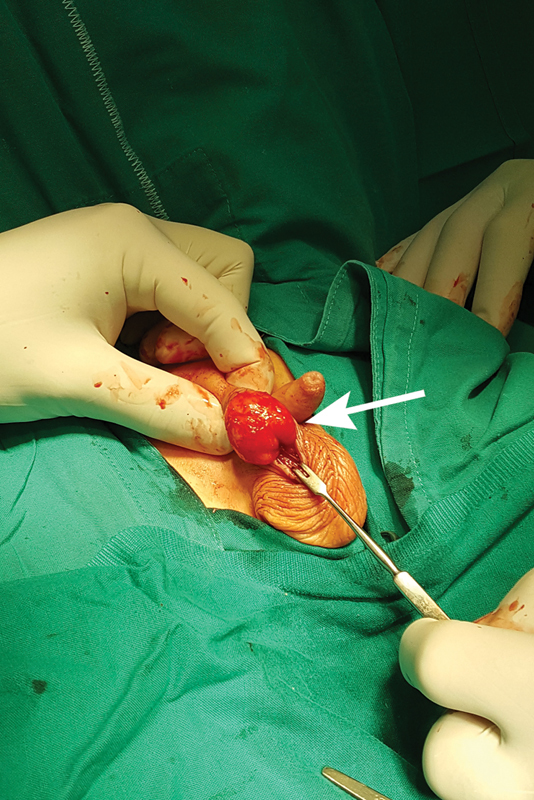
Solid tumor of 3 × 2 cm in size marked by an arrow.

**Fig. 4:**
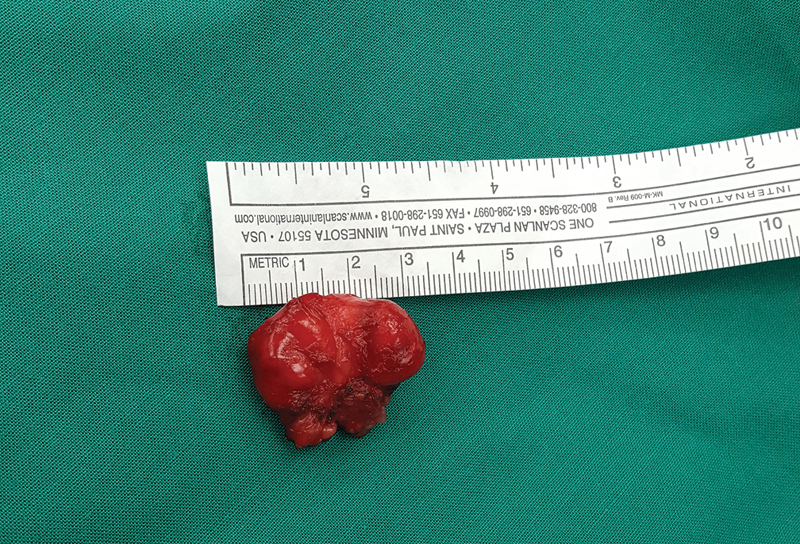
Extirpated tumor of 3 × 2 cm in size.

**Fig. 5:**
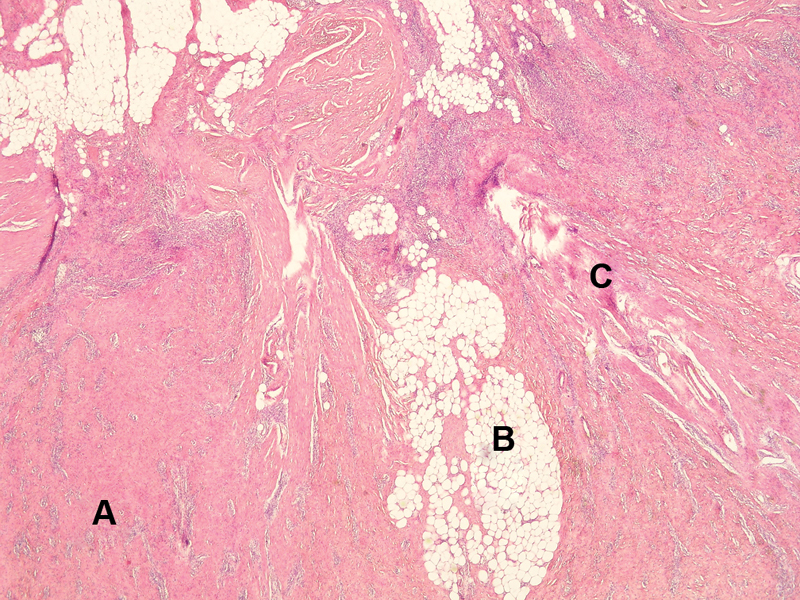
Electron microscope increased ×20 hematoxylin and eosin stain; characteristic components—fibrous connective tissue ( **A** ), islands of mature fat cells ( **B** ), and mesenchymal stroma ( **C** ).

**Fig. 6:**
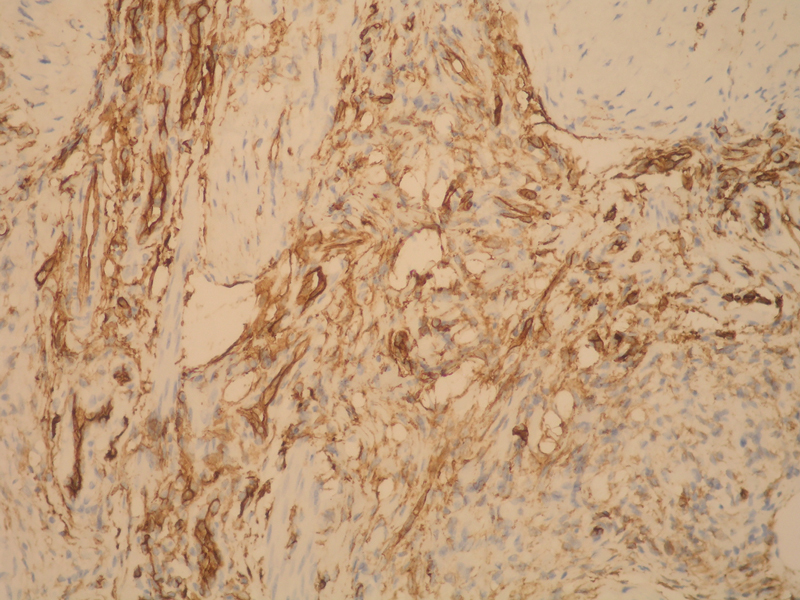
Immunohistochemistry increased ×20; CD34 positive cells inside hyaline stroma.

The first and healthy term child was born to a mother with an unremarkable family history. The mother noticed a formation in the right hemiscrotum when the child was 8 months old. She was not sure if formation changed its size according to daily activities of the child. On examination, a solid, movable, painless formation was verified, 1 cm in diameter, located in the upper half of the right hemiscrotum. Both testicles were descended in the scrotum. The right testicle did not seem connected to the formation. No clear signs of inguinal hernia or testicular hydrocele were found. Regional lymph nodes were not increased. The skin was intact. The child seemed clinically unaffected and the working diagnosis was hydrocele funiculi. Specific laboratory and radiological tests such as ultrasonography, computed tomography (CT) scan, magnetic resonance imaging (MRI), and tumor markers were not done. According to the guidelines of our hospital, the follow-up was after 3 months. However, during a follow-up examination 3 months later, progression was verified. A solid formation was palpated, still located in the upper half of the right hemiscrotum, painless, without symptoms, not connected to the testicle, now ∼2 × 2.5 cm in diameter ( Fig. 2 ). After 3 months, mother was still unsure if the formation changed its size. Working diagnosis was still hydrocele funiculi. Because of the growing mass, the location of the tumor, age of the child, and concern of the parents, the indication for surgery was made. We used scrotal approach. Horizontal incision was made in upper half of right hemiscrotum. During surgery, a well-limited solid formation was found on the external inguinal ring, 3 × 2 cm in diameter, not infiltrating the surrounding structures ( Fig. 3 ). Considering the location of the formation, we decided to use an additional inguinal approach to examine the communication between the formation and the structures of the inguinal canal. The formation was not connected to the elements of the inguinal canal, the spermatic cord, and the testicle. Malignant formation was not verified, enlarged regional lymph nodes were also not verified, and for that reason, open biopsy or urgent histopathological analysis was not done. The tumor was completely removed preserving the surrounding structures ( Fig. 4 ). Histologically characteristic components included well-differentiated fibrous connective tissue, primitive mesenchymal stroma, and islands of mature fat cells. Immunohistochemical analyses (smooth muscle actin, h-caldesmon, desmin, S-100, epithelial membrane antigen, b-catherin, K _i_ -67, Bcl-2, CD 34) confirmed the diagnosis of an FHI ( Figs. 5 and 6 ). Macroscopic resection margins were tumor free, which was later also confirmed by histopathological analysis.

## Discussion

This case demonstrates a rare tumor of the scrotal region which, due to its nonspecific clinical picture, presents a diagnostic and therapeutic challenge in clinical practice. Considering the nonspecific clinical picture, the consistency of the formation, the area it affects, and its tendency to grow, in terms of differential diagnosis, several conditions can be taken into account 7 8 9 10 ( Table 1 ). Out of all diseases, malignant ones deserve special attention. For this reason, according to the literature, every solid formation in the scrotal region which tends to grow is considered as malignant until the opposite is proven. 8 It has been shown that 75% of tumors of the scrotum, including malignant tumors, occur before the second year of life of a child, which correlates with period of occurrence of FHI. 7 Our patient had unspecific clinical presentation and medical history. Guided by this information, during preoperative follow-up period, working diagnosis was hydrocele funiculi. As such, it became indication for operation. In retrospective, after finished treatment, we should have done more extensive tests which would have given us wider perspective, especially on potential malignity. FHI is usually diagnosed up to second year of child's life and in that period, the most frequent tumors of scrotal region are: yolk sac tumors, teratomas, epidermoid cysts, choriocarcinomas, and sarcomas. 9 10 11 12 Ultrasonography is the diagnostic method for scrotal formations. 11 Depending on the ultrasonography findings, it is advisable to extend tests to X-ray, CT scan, and MRI for staging and exclusion of metastasis. 9 11 In laboratory tests, serum for tumor markers, alpha-fetoprotein (AFP), and beta human chorionic gonadotropin are examined. 9 10 11 12 It should be taken into account that AFP is physiologically elevated from 6 to 8 months after birth. 11 12 Since clinically and radiologically, it is not possible to establish the diagnosis of FHI and exclude the possibility of a malignant formation which would require a different approach and treatment, the method of choice is total removal of the formation and definite histopathological analysis. 3 5

**Table 1:** Differential diagnosis of FHI

Neurofibromatosis
Fibrolipoma
Angiolipoma
Juvenile hyaline fibromatosis
Hemangioma
Lymphangioma
Dermoid cyst
Germ cell tumors
Teratoma
Seminoma
Endodermal sinus tumor
Gonadal stromal tumors
Leydig cell
Sertoli cell
Juvenile Granulosa cell
Gonadoblastoma
Rhabdomyosarcoma
Leiomyosarcoma

In most cases, the microscopic finding has three characteristic components: well-differentiated fibrous connective tissue, primitive mesenchymal stroma, and islands of mature fat cells. 2 According to some authors, it is exactly the primitive mesenchymal cells that can be misinterpreted as rhabdomyosarcoma, infantile fibrosarcoma, or infantile myofibromatosis. 2 9 Therefore, establishing the diagnosis is a challenge for a histopathologist as well. It is pointed out in the literature that there is no actual proof of remission of FHI, its metaplasia or malignant dysplasia. 5 In case of incomplete removal, there is a possibility of a relapse of up to 15% with median rate of occurrence in the fifth month from the operation. 1 2 3 5 6 13 In our case, complete removal was done with tumor-free margins. Follow-up was in 6th and 12th months from the procedure using ultrasonography. One year after the procedure, a control ultrasonography examination showed no relapse. Considering that clinical findings and radiology tests were normal, it was concluded that the treatment was finished.

## Conclusion

FHI is a benign and rare formation in the subcutis with a good prognosis. Considering the nonspecific clinical picture, this formation deserves wider diagnostic tests to exclude malignant disease. The method of treatment is complete excision. The final diagnosis is confirmed by histopathological analysis.

## Notes

New Insights and the Importance for the Pediatric Surgeon This case report is significant for the pediatric surgeon because it presents a rare benign tumor which is presented by a nonspecific clinical picture and diagnostic findings according to which it is not possible to exclude the possibility of a malignant formation. For that reason, we believe it is important to present this work, to demonstrate the treatment approach and the outcome, which is significantly different from those related to a malignant formation.
